# Custom-Developed Reflection–Transmission Integrated Vision System for Rapid Detection of Huanglongbing Based on the Features of Blotchy Mottled Texture and Starch Accumulation in Leaves

**DOI:** 10.3390/plants12030616

**Published:** 2023-01-30

**Authors:** Qian Xu, Jianrong Cai, Lixin Ma, Bin Tan, Ziqi Li, Li Sun

**Affiliations:** School of Food and Biological Engineering, Jiangsu University, Zhenjiang 212013, China

**Keywords:** Huanglongbing, classification, image acquisition, pattern recognition, classifier

## Abstract

Huanglongbing (HLB) is a highly contagious and devastating citrus disease that causes huge economic losses to the citrus industry. Because it cannot be cured, timely detection of the HLB infection status of plants and removal of diseased trees are effective ways to reduce losses. However, complex HLB symptoms, such as single HLB-symptomatic or zinc deficiency + HLB-positive, cannot be identified by a single reflection imaging method at present. In this study, a vision system with an integrated reflection–transmission image acquisition module, human–computer interaction module, and power supply module was developed for rapid HLB detection in the field. In reflection imaging mode, 660 nm polarized light was used as the illumination source to enhance the contrast of the HLB symptoms in the images based on the differences in the absorption of narrow-band light by the components within the leaves. In transmission imaging mode, polarization images were obtained in four directions, and the polarization angle images were calculated using the Stokes vector to detect the optical activity of starch. A step-by-step classification model with four steps was used for the identification of six classes of samples (healthy, HLB-symptomatic, zinc deficiency, zinc deficiency + HLB-positive, magnesium deficiency, and boron deficiency). The results showed that the model had an accuracy of 96.92% for the full category of samples and 98.08% for the identification of multiple types of HLB (HLB-symptomatic and zinc deficiency + HLB-positive). In addition, the classification model had good recognition of zinc deficiency and zinc deficiency + HLB-positive samples, at 92.86%.

## 1. Introduction

Citrus is one of the most popular fruits in the world, with a total production of 144 million tons worldwide, according to the Food and Agriculture Organization of the United Nations [1]. In the process of citrus cultivation, it will be harmed by pests and diseases, among which Huanglongbing (HLB) has the most serious impact. The culprit for the severe damage caused to the citrus industry is the psyllid-spread phloem-limited bacterium “*Candidatus* Liberibacter asiaticus” (CLas) [2,3]. The most serious consequences are the loss of the edible value of fruit and the death of citrus trees. In addition, HLB cannot be cured, and the proliferation of HLB has a great impact on the citrus industry [4]. Achieving accurate detection of HLB and removing diseased trees are effective ways to reduce losses [5].

Traditional methods for the detection of HLB include field diagnosis, indicator crop identification, microscopic pathogen observation, serological identification, hybrid criteria, DNA probe hybridization, polymerase chain reaction (PCR), and quantitative real-time polymerase chain reaction (qPCR) [6]. The field diagnosis method relies on the extensive experience of the inspector; despite this, its accuracy rate is less than 70% [7]. Other methods require the support of expensive equipment, and the detection process is tedious and time-consuming. Currently, PCR and qPCR are the most commonly used methods to confirm HLB infection [8]. However, due to their high cost, it is not possible to apply them to large-scale orchards, and in practice they still rely on human screening of suspicious leaves for detection.

In recent years, many fast and effective methods have been developed to detect HLB as quickly as possible. Yellow shoots and blotchy mottles are the common symptoms of HLB-affected plants [3,9]. For these symptoms, HLB detection methods based on machine vision and pattern recognition have been proposed. A customized fluorescence imaging system was used for the acquisition of images of citrus leaves [10,11]. In this study, the SVM and ANN obtained 92.8% and 92.2% accuracy, respectively. Deng et al. used natural light to acquire citrus leaf images, and C-SVC and BPNN were used to perform pattern recognition, with an accuracy of 91.93% and 92%, respectively [6,12]. Liu et al. used hyperspectral imaging to acquire citrus leaf images and extracted image features based on GLCM [13]. The PLS-DA model was used for the identification of HLB-symptomatic leaves, and the results showed that the method was effective for the identification of HLB-symptomatic samples.

HLB infection manifests as phloem necrosis, which is thought to be the main reason for the accumulation of starch in leaves, because it prevents photosynthetic products from being transported to the roots [14]. The leaves in a healthy state produce only trace amounts of starch, which can be neglected compared to the amount of starch accumulated in HLB-affected leaves [9]. The abnormal accumulation of starch is another typical feature of HLB infection. On this basis, Pourreza et al. conducted extensive and thorough studies and obtained a high confidence level [15,16]. However, the smooth and thick waxy layer on citrus leaves hinders the entry of polarized light, reducing the potential for detecting the optical activity of starch in leaves by optical imaging. In addition, the image acquisition method of reflection imaging confuses the yellowing areas caused by nutrient deficiency with those caused by starch accumulation. Meanwhile, both zinc deficiency and HLB can cause abnormal accumulation of starch in the leaves [17], but the difference is in the form of starch accumulation and the content of starch [18,19]. The immediate effect of zinc deficiency seems to be to prevent the export of photosynthetic products from the chloroplasts, causing the accumulation of starch in the chloroplasts and resulting in their destruction [20]. The accumulation of starch in zinc-deficient leaves occurs within the chloroplasts, and almost all chloroplasts are affected, so the yellowing areas are evenly distributed between the leaf veins. HLB causes blockage of the phloem and, subsequently, the translocation of starch from the leaves to the roots is blocked and starch granules accumulate in the leaves in large quantities, causing crushing and destruction of the chloroplasts’ structure [9]. This destruction is irregular, so the leaves show mottled yellowing. Differences in starch accumulation patterns result in differences in leaf symptoms between zinc-deficient and HLB-positive samples. However, in the later stages of HLB infection, zinc deficiency symptoms usually accompany HLB symptoms, and the symptoms of HLB are masked by zinc deficiency symptoms [3]. In summary, the information in the images obtained by a single reflection imaging method is limited and cannot be adapted to the complex and diverse symptoms of HLB in the natural environment. Therefore, it is more likely to achieve the detection of multiple types of HLB by considering two typical symptoms of HLB infection—blotchy mottles and abnormal accumulation of starch—in an integrated manner. The objectives of this study were as follows:

1. To develop a device for real-time HLB detection in the field.
2. To develop an imaging system capable of highlighting the blotchy, mottled texture of HLB-affected leaves in reflection imaging mode.
3. To develop a method capable of calculating a polarization angle image that can reflect the relative starch content and distribution within the leaf in transmission imaging mode.
4. To develop an optimal step-by-step classification model for the detection of multiple types of HLB-affected samples (HLB-symptomatic and Zn deficiency + HLB-positive).

## 2. Results

### 2.1. Results of Image Acquisition and Pre-Processing

**Figure 1:**
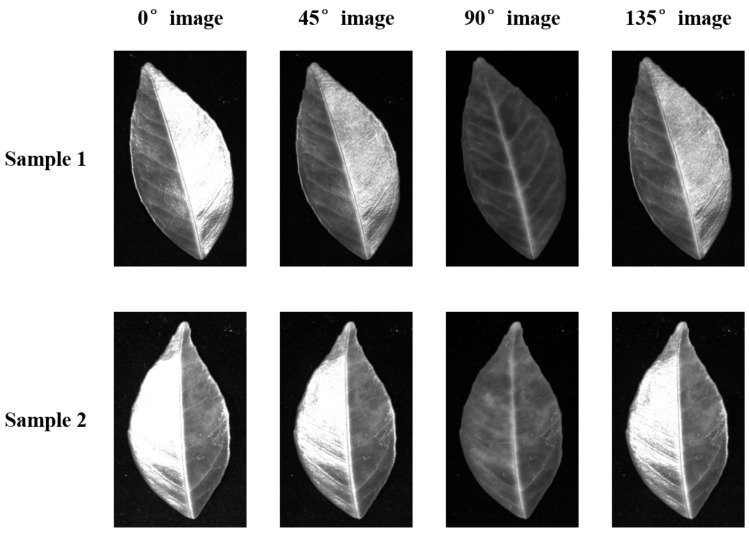
Examples of four polarization angle images acquired in reflection imaging mode.

Figure 1 shows examples of four polarization angle images acquired in reflection imaging mode combined with polarized light imaging. The 0°, 45°, and 135° images all had significant leaf surface reflections, which obscured the symptoms of the leaves. The 90° images presented the symptoms of the leaves and were selected for pre-processing in reflection imaging mode. This method based on polarized light imaging to separate reflection components has been proposed and applied in the field of computer vision [21].

**Figure 2:**
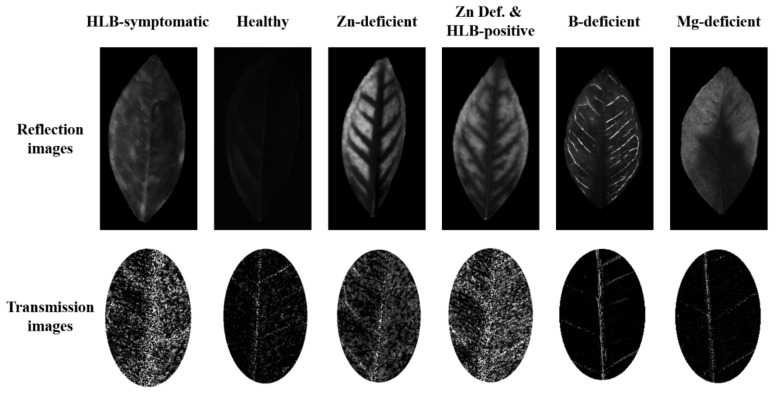
Example images of different types of leaves acquired using the custom-developed vision system.

Figure 2 shows the pre-processed reflection and transmission images representing the six types of leaf samples involved in this study: HLB-symptomatic, healthy, Zn deficiency, Zn Def. + HLB-positive, B deficiency, and Mg deficiency. The absorption spectrum of chlorophyll has the strongest absorption peak near 660 nm (red light region) [22]. Therefore, the reflection image generated by the 660 nm illumination has a strong suppression effect on the imaging of chlorophyll. In the reflection image, there was no chlorophyll loss in healthy leaves, resulting in light at 660 nm being absorbed by chlorophyll and, therefore, showing a continuous black area. HLB-symptomatic samples showed discontinuous, mottled yellowing because of the random destruction of chlorophyll within the leaves due to starch accumulation [9]. The Zn- and Mg-deficient samples showed a symmetrical yellowing pattern, which was different from the yellowing caused by HLB [3]. Zn deficiency often accompanies HLB infection in its later stages [3], and it is difficult to distinguish HLB infection in Zn-deficient leaves based on reflection images because Zn deficiency symptoms mask HLB symptoms [15]. B deficiency leads to lignification of the xylem cell walls [23], so the veins were more pronounced in images of B-deficient citrus leaves, as shown in Figure 2.

Transmission images were used to represent the content and distribution of starch in the leaves. Compared to the HLB-affected samples, no appreciable amount of starch was accumulated in the healthy, Mg-deficient, and B-deficient samples, so the differences in the transmission images were not significant. As shown in Figure 2, samples can be divided into two categories according to the presence or absence of significant starch accumulation. Among them, samples with no significant accumulation of starch—such as healthy, Mg-deficient, and B-deficient samples—had low grayscale values in the mesophyll and high grayscale values in the leaf veins. Samples with significant starch accumulation—such as HLB-symptomatic, Zn-deficient, and Zn Def. + HLB-positive samples—had large numbers of high-brightness pixel points in the mesophyll. Similar to the HLB-symptomatic samples, the Zn-deficient samples also showed chlorophyll loss due to starch accumulation, except that Zn deficiency prevented the transfer of photosynthetic products from the chloroplasts and caused starch to accumulate uniformly in all chloroplasts, whereas HLB caused uneven blockage of the phloem, so the chlorophyll loss due to Zn deficiency was more uniform and symmetrical. The starch content of Zn-deficient leaves was higher than that of healthy leaves but lower than that of HLB-symptomatic leaves [15,24], which is consistent with the results of the images that we obtained. In addition, the transmission images of Zn Def. + HLB-positive samples had more pixels than the Zn-deficient and HLB-symptomatic samples, so we speculated that the starch content in Zn Def. + HLB-positive samples was higher than that in HLB-symptomatic and Zn-deficient samples. The presence of high-brightness pixels in the veins of all samples was caused by the inability of light to penetrate the veins. Specifically, the leaf vein surface was illuminated by the light passing through the mesophyll and captured by the camera, so high-brightness pixels were displayed in the images acquired in transmission imaging mode. However, the difference in the distribution of pixel points in the mesophyll was sufficient to identify the presence and extent of starch accumulation in leaves; therefore, highly luminous pixels of leaf veins had an acceptable impact on the classification process.

### 2.2. Results of the Step-by-Step Classification Model

Compared to the interference of leaf veins’ highlighted pixels in the transmission images, the reflection images were subjected to less interference and had excellent differentiation ability, except for Zn-deficient and Zn Def. + HLB-positive samples. Accordingly, a step-by-step classification model was designed. The samples were classified into five categories using reflection imaging mode: healthy, Mg deficiency, B deficiency, HLB-symptomatic, and Zn deficiency broad category (Zn-deficient and Zn Def. + HLB-positive samples), and the subdivision of the Zn deficiency broad category was performed using the transmission imaging mode.

Table 1 shows the accuracy of the five classifiers at each step in the classification model, and the best combination of classifiers was selected accordingly. Among them, the LR had good classification results in the first, second, and fourth steps, LDA also had good classification effects in the second step, and RF had the best classification effects in the third step.

The complete detection process was performed on the dataset of all categories of samples using the best classifier in each step of the classification model, and the step-by-step classification results are shown in Table 2. The average identification rate of the model for the six categories of samples was 96.92%, with 100% for healthy, Mg-deficient, B-deficient, and HLB-symptomatic samples. In the third step of the classification model, three Zn Def. + HLB-positive samples were incorrectly identified as HLB-symptomatic samples. These three samples could have been accurately identified in the fourth step, which introduced errors in the identification of Zn Def. + HLB-positive samples. This is an unavoidable problem for step-by-step classification models, because the identification in the next step is based on the identification in the previous step and, thus, the risk of misclassification in the previous step needs to be taken. However, if the classifier of each step is reliable enough, then this risk will be greatly reduced. In this study, this error could be acceptable considering the high accuracy of the step-by-step model.

The main objective of this study was to identify multiple types of HLB, so all categories of samples were classified into two categories (HLB-positive and HLB-negative); the classification results are shown in Table 3. The average classification accuracy of the model was 98.08% when only the recognition rate of HLB was considered.

## 3. Materials and Methods

### 3.1. Citrus Leaves Collection

Citrus leaves were acquired from Newhall Gannan navel orange trees in Ganzhou Citrus Scientific Research Institute (Ganzhou, Jiangxi Province, China) in November 2021 under the guidance of experienced horticulturalists. Six kinds of leaf samples—healthy, HLB-symptomatic, zinc deficiency (Zn deficiency), samples of the combined effect of Zn deficiency and HLB (Zn Def. + HLB-positive), magnesium deficiency (Mg deficiency) and boron deficiency (B deficiency)—were collected. All samples were packaged in Ziploc moisture barrier packets, transported to the laboratory via cold chain, and subsequently tested for HLB by qPCR [8]. A total of 260 leaf samples were tested, including 50 healthy samples, 50 HLB-symptomatic samples, 50 Zn-deficient samples, 20 Zn Def. + HLB-positive samples, 50 Mg-deficient samples, and 40 B-deficient samples.

### 3.2. Custom-Developed Vision System

**Figure 3:**
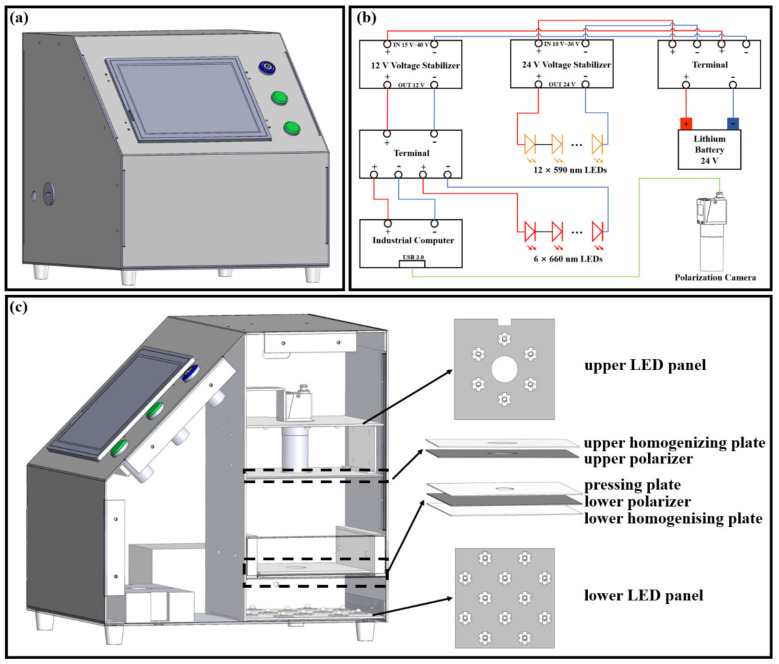
Custom-developed vision system: (**a**) appearance of the vision system; (**b**) circuit diagram of the power supply module; (**c**) schematic diagram of the image acquisition module.

Considering the complex symptoms of HLB, combined with the internal and external symptoms (i.e., mottled leaves and starch accumulation) of the HLB-affected leaves, using these two imaging modes for HLB detection is the better choice. This approach was used to design the vision system, which was packaged in a galvanized sheet box (30 cm × 30 cm × 30 cm) including the image acquisition module and the power supply module (Figure 3). A touchscreen industrial computer was used for the operation of the entire identification process and to display the results. The industrial computer was powered by a DC power supply (12 V, 5 A) and equipped with a USB interface. As shown in Figure 3b, the 24 V lithium battery was stepped down by a 12 V voltage stabilizer to supply power to the industrial computer.

The illumination system of the image acquisition module was divided into two parts: reflection mode and transmission mode, which were used to detect the mottled symptoms of citrus leaves and the internal starch accumulation, respectively. The massive accumulation of starch induced by HLB leads to the destruction of chloroplasts’ structure, of which the most direct external expression is the yellowing of the leaves [14]. To highlight this property, six LEDs (LED Engin, San Jose, CA, USA) at 660 nm (LZ1-10R202-0000, 2.5 W) were used to provide illumination for the reflection imaging mode. The reflection rate of light was higher for lutein and carotenoids, and the absorption rate was highest for chlorophyll under 660 nm LED light source irradiation [25]. This helped to highlight the regions of leaves with mottled symptoms in the images. Starch has the property of rotating the polarization plane of polarized light, and a 589.6 nm wavelength is usually used to detect this optical activity [18]. To avoid the blocking of polarized light by the waxy layer on the leaf surface, the transmission imaging mode was used to measure the ability of starch to rotate the polarization plane of light. In addition, 12 LEDs (Cree LED, Durham, NC, USA) at 590 nm (XPEBAM-L1-0000-00902, 3 W) were used to provide illumination for the transmission imaging mode. As shown in Figure 3b, all of the LEDs were powered by a 24 V lithium battery after being regulated by a 24 V voltage stabilizer.

Figure 3c shows the schematic diagram of the custom-developed vision system. The image acquisition module was sealed in a space of 15 cm × 15 cm × 30 cm to obtain a single illumination source and avoid reflections from other objects. The vision system used a grayscale polarization camera (MER-502-79U3M POL, Daheng Imaging, Beijing, China) as the image acquisition machine. In addition, the polarization camera could be used for data transfer and power supply via a USB interface. The camera was equipped with a Sony IMX250MZR CMOS (IMX250MZR, Sony, Tokyo, Japan) polarization sensor. It had a resolution of 5 megapixels and a frame rate of 79 fps. Four polarizers with different polarization directions (0°, 45°, 90°, and 135°) were integrated inside the camera to acquire four polarized images. The camera was fitted with a lens (M0824-MPW2, Computar, Tokyo Japan) with a focal length of 8 mm, the purpose of which was to reduce the size of the device for portability.

To improve the imaging quality, linearly polarized light was used to provide illumination for the reflection imaging mode. In the transmission imaging mode, the leaves were flattened using a pressing plate with an elliptical hole in the center to obtain a flat transmission image, and linearly polarized light was used to generate polarized images of the leaves. To ensure uniformity of the light, homogenizing plates were used. All of the linearly polarized light was generated through the polarizer in front of the illumination source. The polarization directions of all of the polarizers coincided with the polarization direction of the 0° polarizer in the polarization camera. Therefore, four images were acquired in each of the reflection and transmission imaging modes:

1. 0° image: generated by polarized light and the 0° polarizer in the polarization camera.
2. 45° image: generated by polarized light and the 45° polarizer in the polarization camera.
3. 90° image: generated by polarized light and the 90° polarizer in the polarization camera.
4. 135° image: generated by polarized light and the 135° polarizer in the polarization camera.

### 3.3. Image Pre-Processing

**Figure 4:**
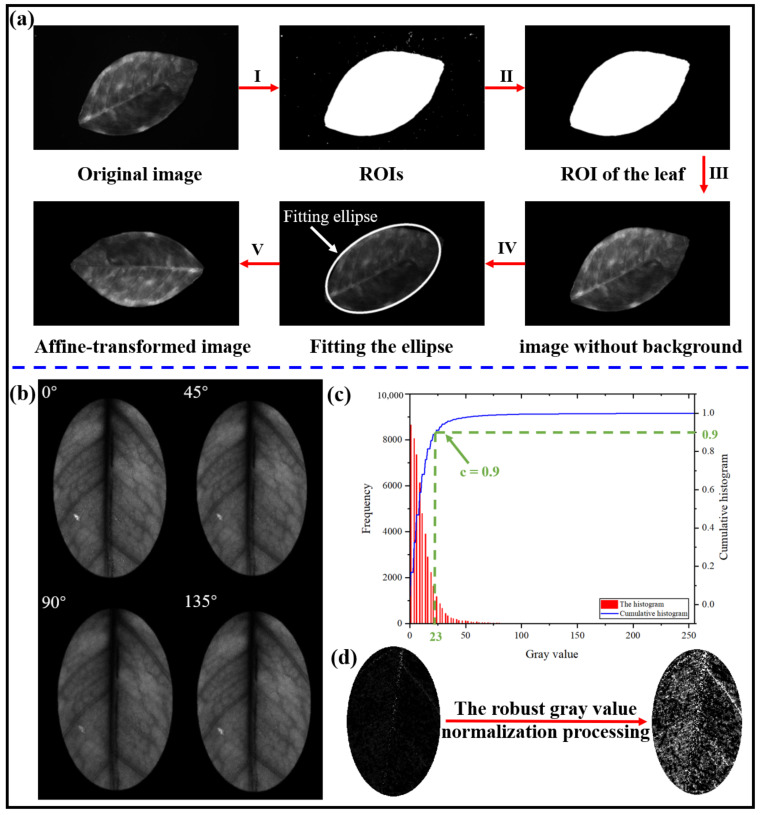
Pre-processing of leaf images in two imaging modes: (**a**) the pre-processing of the reflection image: (I) thresholding, (II) extraction of connected components, (III) acquiring leaf images of the region of interest (ROI), (IV) fitting the ellipse, and (V) affine transformations; (**b**–**d**) the pre-processing of the transmission image: (**b**) the original image obtained in transmission imaging mode, (**c**) histogram of the robust gray value normalization process, and (**d**) pre-processing result of the transmission image.

The 90° image was chosen to perform the pre-processing for reflection imaging because it had proven to be effective in improving the image quality (as explained later in the Section 2). The flow of the pre-processing method in the reflection imaging mode is shown in Figure 4a, which aimed to remove the background in the image and make the orientation of the leaves uniform. The purpose of removing the background in the image was to simplify the image and remove the interference of external substances in feature extraction; the purpose of making the orientation of the leaves uniform was to facilitate the extraction of texture features. The pre-processing consisted of the following five steps:

- Triangular threshold segmentation was used in separating the image background [26].
- The maximum connected component was extracted to obtain the leaf region.
- To obtain the background-free image of the leaf, the original image was multiplied by the leaf region.
- The ellipse was fitted according to the blade shape.
- The rotation angle in the affine transformation was determined according to the angle between the long axis of the fitted ellipse and the horizontal direction, and all of the leaf images were changed to the horizontal position.

In the transmission imaging mode, four polarization images were obtained, as shown in Figure 4b. To obtain a clearer picture of the changes in the polarization state of light occurring due to the starch inside the leaf, the polarization state of the light was described using the Stokes vector, which is often applied for the mathematical description of polarized light states [27]. Stokes’ theory applies to both partially and fully polarized light. Due to the anisotropy of the citrus leaf, polarized light crossing the leaf becomes a combination of polarized and unpolarized light, and the Stokes vector can perfectly represent the polarization characteristics of the light after crossing the leaves. The Stokes vector contains four Stokes polarization parameters (*S*_0_, *S*_1_, *S*_2_, and *S*_3_), where *S*_0_ represents the intensity of the whole light and *S*_1_, *S*_2_, and *S*_3_ represent independent polarization states in different directions. The formula is as follows:

(1) $$S=\left(\begin{array}{c} S_{0}\\ S_{1}\\ S_{2}\\ S_{3} \end{array}\right)=\left(\begin{array}{c} I\\ Q\\ U\\ V \end{array}\right)=\left(\begin{array}{c} I_{0}+I_{90}\\ I_{0}-I_{90}\\ I_{45}-I_{135}\\ I_{R}-I_{L} \end{array}\right)$$

where *I* is the sum intensity value of light, *Q* is the subtraction of light intensity for the 0° and 90° images, *U* is the subtraction of light intensity for the 45° and 135° images, and *V* is the intensity subtraction of the beam in the left and right circularly polarized light components.

Since starch has the property of rotating the plane of polarized light, different concentrations of starch lead to different angles of rotation of the plane of polarized light, so the angle of linear polarization (*AoLP*) was determined using the Stokes vector method to describe the differences in the content and distribution of starch within the leaves. In general, the *AoLP* is a valid indicator to describe polarization characteristics in polarization imaging studies [28]. The *AoLP* represents the angle between the direction of the vibration of light and the reference direction, which can characterize the angle of rotation of the polarization plane after the polarized light passes through the citrus leaf, as described in the following equation:

(2) $$AoLP=\frac{1}{2}\arctan\left(\frac{U}{Q}\right)$$

The polarization angle image calculated by Equation (2) is shown in Figure 4d, where the trace amount of starch in the leaves led to a lower contrast in the polarization angle image. The robust gray value normalization method [29] was used to enhance the contrast of the image with the following equations:

(3) f(g)=g*Mult+Add,f(g)∈[0,255]

(4) f(g)=Min(Max(⌊g*Mult+Add+0.5⌋,0),255),f(g)∉[0,255]

(5) $$Mult=\left\lfloor \frac{255}{g_{\mathrm{Max}}-g_{\mathrm{Min}}}\right\rfloor$$

(6) $$Add=-Mult*g_{\mathrm{Min}}$$

(7) $$c_{i}=\sum_{j=0}^{i}\frac{n_{j}}{n}$$

where *g* represents the gray value of the original image, *Mult* represents the multiplication factor, *Add* represents the summation factor, *f(g)* represents the gray value of the image after enhancement, *g_Max_* is the maximum gray value, *g_Min_* is the minimum gray value, *c_i_* is the cumulative probability of grayscale values, *n_i_* denotes the number of pixels with gray value *i*, and *n* is the number of pixels in the region of interest (ROI). To prevent *f(g)* from going outside the range of gray values (i.e., 0 to 255), Equation (4) was used for cropping. *Mult* and *Add* control the changes in the image’s contrast and brightness, respectively. The normal gray value normalization method directly determines the maximum and minimum gray values in the image as *g_Max_* and *g_Min_*, respectively. However, this gray value normalization method is useless if the darkest (gray value = 0) and brightest (gray value = 255) points are in the image. The robust gray value normalization method used in this study determines *g_Max_* and *g_Min_* by counting the cumulative histogram of the image and setting the cumulative histogram threshold to filter the gray value of the image. For example, as shown in Figure 4c, there were pixel points with gray values equal to 0 and 255 in the image, and the cumulative probability was greater than 90% in the region where most of the image’s gray values were concentrated. The cumulative probability threshold was set to 0.9, *g_Max_* = 23 and *g_Min_* = 0 were determined, and *Mult* = 11 and *Add* = 0 were calculated. The before-and-after comparison of image enhancement is shown in Figure 4d.

### 3.4. Feature Extraction

The 90° image in reflection imaging mode and the enhanced polarization angle image calculated using the four polarization images obtained in transmission imaging mode were used for feature extraction. Texture features extracted from the gray level co-occurrence matrix (GLCM) and statistical histogram features of gray values (mean and standard deviation) were extracted.

The GLCM is a common method used in image feature extraction, showing the frequencies of two different grayscale values adjacent to one another in four main directions (0°, 45°, 90°, and 135°) [15]. The normalized GLCM model proposed by Gómez et al. was used in this study [30]. Usually, some scalars were used to characterize the GLCM. In this study, four features were selected to characterize the GLCM, including Energy, Correlation, Homogeneity, and Contrast, as shown in Table 4. The mean and standard deviation of the values of these four features in the four directions of 0°, 45°, 90°, and 135° were calculated and, finally, eight texture features were obtained to represent one leaf sample.

Two statistical histogram features—the mean and standard deviation of the gray values—were calculated based on the ROIs of the leaf images. The calculation formula is shown in Table 4 (Equations (12) and (13)), where *R* is the ROI of the image, *p* is the pixel point of *R*, *g(p)* is the gray value, and *F* is the number of pixels in *R*.

### 3.5. Classification Models

**Figure 5:**
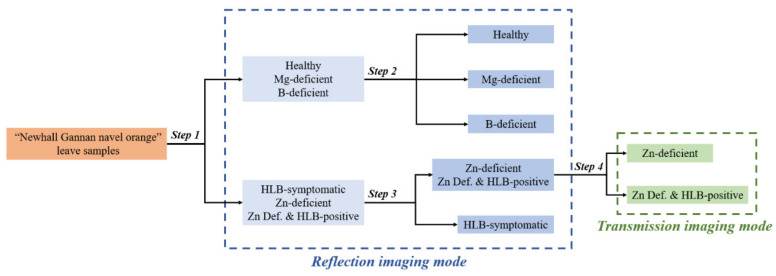
Schematic diagram of the step-by-step classification model.

Based on the findings of our preliminary experiments and the texture differences between different classes of leaves, an optimal step-by-step classification model was developed. The aim was to simplify the operation, convert the complex classification into a simple dichotomous classification, and improve the classification performance by making full use of the respective advantages of reflection imaging and transmission imaging. The step-by-step classification model is shown in Figure 5, which includes a total of four classification steps and, ultimately, divides the samples into six subclasses. The first, second, and third of these steps used the reflection imaging mode, while the fourth step used the transmission imaging mode. In the process of determining the best classifier for each step, the dataset was divided into two parts—the training set and the test set—at a ratio of 7:3 (70% training data and 30% test data).

To select the optimal pattern recognition method, the classification performance of five pattern recognition methods—linear discriminant analysis (LDA), random forests (RF), support-vector machine (SVM), classification and regression tree (CART), and logistic regression (LR)—was evaluated in each classification step.

LDA is a classic supervised dimensionality reduction classification technique, which is usually used for the dimensionality reduction and classification of data. Since LDA gives the data the largest interclass distance and the smallest intraclass variance in the new dimensional space, the distinguishability of the reduced-dimensional sample data is better than that of the original data [31]. RF aggregates multiple weak classifiers into strong classifiers and uses multiple trees to differentiate and classify the data. Improving prediction accuracy without significantly increasing computational effort is a significant advantage of RF [32]. SVM is a dichotomous classification model whose basic model is a linear classifier with a maximum interval defined in the feature space, which solves the decision boundary of the maximum bounded hyperplane by learning samples [33]. CART simplifies computation by repeatedly classifying or regressing binary data. Patterns of values of different input and output variables are obtained through data learning, data classification, and prediction. These patterns are then used to classify and predict new data objects. The decision tree infers the categorical values of the output variables based on the values of the input variables of the new data. LR assumes that the data obey Bernoulli distribution, and it uses gradient descent to solve the parameters by maximizing the likelihood function to achieve the purpose of dichotomizing the data. Standard logistic regression uses a weighted linear combination of the coefficients of the input variables to classify the data. Accuracy (i.e., the ratio of the number of correctly classified samples to the total number of samples) was used to evaluate the classification performance of the models, and the classifier with the highest average accuracy in the training and test sets at each step was considered to be the best classifier.

After determining the best classifier for all steps, each dataset was randomly divided into two equal sets. The entire model was then run with one set as the training set and the other set as the validation set. Then, the classification model was repeated with the two sets in swapped roles. Thus, classification results were obtained for all samples.

## 4. Discussion

In this paper, a portable custom-developed vision system equipped with two imaging modes was developed to distinguish multiple types of HLB-affected samples (HLB-symptomatic and Zn Def. + HLB-positive) based on the blotchy mottled texture features of the leaves and the abnormal starch accumulation inside the leaves.

Our findings suggested a new approach for the identification of Zn-deficient and Zn Def. + HLB-positive samples, which was achieved using a transmission imaging system equipped with a 590 nm polarized light illumination unit. In the early stages of HLB infection, blotchy mottling may be the only leaf pattern visible. In later stages, symptoms of Zn deficiency will eventually develop, and the HLB-induced Zn deficiency is indistinguishable from genuine Zn deficiency in the leaf symptoms [3]. HLB symptoms can be masked by Zn deficiency symptoms, so the texture features on the leaves cannot be used to accurately distinguish between the Zn-deficient and Zn Def. + HLB-positive samples. Significant differences in starch content between HLB and Zn-deficient samples have been confirmed [17]. This was consistent with the results of the transmission images that we acquired (Figure 2). In addition, our results showed significant differences in the number and distribution of pixel points in the transmission images of Zn-deficient and Zn Def. + HLB-positive samples, as shown in Figure 2, indicating the differences in the content and distribution of starch between them. This difference was used for classification, and the confusion matrix is shown in Table 5. The accuracy of the model for the identification of Zn-deficient and Zn Def. + HLB-positive samples was 92.86%, with five samples being incorrectly identified. The detection of starch within citrus leaves by reflection imaging mode has been reported [16], but this approach is more disturbed by yellowing caused by non-starch accumulation, because the symptoms of starch accumulation and nutrient deficiency are very similar. The method proposed in our study used transmission imaging mode to acquire polarization images of multiple angles, combined with the Stokes vector method to calculate the polarization angle images of leaves [26]. The optical activity of starch within the leaves was detected intuitively, and the interference of yellowing caused by waxy layer reflection and non-starch accumulation was avoided. This method is effective for the identification of Zn-deficient and Zn Def. + HLB-positive samples.

In this study, it was found that the use of a homemade HLB detection device equipped with both transmission and reflection imaging modes could identify classic HLB symptoms and HLB symptoms confused by Zn deficiency symptoms (HLB-symptomatic and Zn Def. + HLB-positive), with the potential for early detection of HLB. The narrow-band polarized light reflection imaging technique used by Pourreza et al. has been used to conduct numerous studies on the optical activity of starch within leaves [15,24], and these studies have made a great contribution to the field of HLB detection. However, the high similarity between starch accumulation regions and nutrient deficiency symptom regions results in the inability to avoid the interference of non-HLB yellowing regions in the reflection imaging mode. Therefore, considering the complexity and variability of HLB symptoms, it is almost impossible to identify HLB-symptomatic and Zn Def. + HLB-positive samples using a single reflection imaging method. In our research, the combined image acquisition method of transmission imaging and reflection imaging was confirmed to have great promise in the field of HLB detection because it can acquire both internal and external features of leaves. Two changes in HLB-affected leaves can be detected: the first is the blotchy mottled texture on the leaves’ surface, and the second is the abnormal accumulation of starch inside the leaves [3,9,14]. Accordingly, we designed two imaging modes for detecting these two symptoms. The reflection imaging mode was designed based on the differences in the light absorption rates of individual components within the leaves at selected wavelengths of light [22], so as to make the disease-affected areas of the leaves more prominent in the reflection images. The transmission imaging mode was used to detect abnormal accumulation of starch within the leaves, and for this purpose a suitable narrow-band illumination system was customized for detecting the optical activity of starch [18,34]. The polarization images acquired by the vision system in four directions were used to calculate the polarization angle images of the leaves by the Stokes vector method. The relative content and distribution of starch in the leaves were determined according to the number and distribution of pixel points in the polarization angle images. The step-by-step classification model shown in Figure 5 was designed to combine the advantages of reflection imaging mode and transmission imaging mode. Ultimately, accurate identification of HLB-symptomatic and Zn Def. + HLB-positive samples, as well as some nutrient-deficient samples, was achieved. As shown in Table 2, the identification accuracy of the model for all categories of samples was 96.92%. Among them, the recognition rate was 98.08% for HLB (HLB-symptomatic and Zn Def. + HLB-positive samples were included). It has been demonstrated that starch accumulation symptoms appear at an earlier stage than blotchy–mottled symptoms [35]. In addition, the feasibility of early detection of HLB was confirmed by Pourreza et al. through optical imaging techniques to detect the optical activity of starch in leaves [36]. The yellowing mottled symptoms of HLB-affected leaves are believed to result from the disintegration of the chloroplast thylakoid system caused by the starch buildup [9]. The mottling symptoms on HLB-affected leaves become more pronounced as the disease progresses. Therefore, the sensitivity of our detection system to mottling symptoms and starch accumulation may be useful for the early detection of HLB, especially in the period when starch starts to accumulate in HLB-affected leaves but does not show symptoms that are visible to the human eye.

Highly accurate laboratory-based methods, such as qPCR detection, require a significant investment in specialized personnel and equipment [8]. In contrast, vision-based detection methods are simpler and more efficient. In our study, a portable and efficient HLB detection method was proposed to achieve the identification of HLB-symptomatic samples as well as samples with a combined effect of nutrient deficiency and HLB. A highly integrated custom-developed detection device was used for rapid detection in the field, and it took only a few seconds to determine the HLB infection status of the samples. The accuracy was acceptable compared to the time-consuming and tedious PCR method. The portable design and the fast and accurate detection results make it possible to use this custom-developed vision system commercially.

## 5. Conclusions

The object of this paper was to use a custom-developed, highly integrated portable vision system for the identification of multiple types of HLB-affected samples (HLB-symptomatic and Zn Def. + HLB-positive). The conclusions are as follows:

1. A portable vision system integrating reflection and transmission imaging modes was designed for the identification of HLB. The custom-developed vision system contained a power module and identification software equipped with the step-by-step classification model, which made it possible to use the equipment in the field.
2. Two internal and external symptoms caused by HLB—(1) the external symptom of blotchy mottled leaves and (2) the internal symptom of abnormal accumulation of starch in leaves—were used for the identification of HLB infection status.
3. A wavelength of 660 nm was used as the illumination band for the reflection imaging mode, where the contrast of the blotchy–mottled symptomatic areas of the HLB-affected samples was enhanced by the light. A wavelength of 590 nm is commonly used for the detection of optical activity; therefore, it was selected as the illumination band for the transmission imaging mode. In combination with the Stokes vector method, the polarization angle image was calculated to visualize the extent to which the plane of polarized light was rotated by the starch, and this was used to infer the distribution of starch in the leaf.
4. A step-by-step classification model was developed based on the respective advantages of the transmission and reflection imaging modes. The recognition accuracy of six types of samples (HLB-symptomatic, healthy, Zn deficiency, Zn Def. + HLB-positive, B deficiency, and Mg deficiency) was 96.92%, including 98.08% for HLB-symptomatic and Zn Def. + HLB-positive samples. In addition, a novel method was developed for the detection of Zn Def. + HLB-positive samples, with a recognition rate of 92.86%.
5. In this study, we achieved the identification of HLB-symptomatic and Zn Def. + HLB-positive samples, along with some nutrient-deficient (i.e., Zn deficiency, B deficiency, and Mg deficiency) samples, with high accuracy. In addition, the low-cost (approximately USD 1500) integrated design allows for a custom-developed vision system that is expected to be commercially available within the next three years, which will be important for HLB prevention.

## Figures and Tables

**Table 1:** The best set of classifiers for each step of the classification models.

Step No.	Imaging Mode	Accuracy (%)					Best Classifier
		LDA	RF	SVM	CART	LR	
1	Reflection	98.85	99.62	98.46	94.23	100.00	LR
2	Reflection	100.00	96.43	98.57	90.00	100.00	LDA, LR
3	Reflection	96.67	97.50	95.83	94.17	96.67	RF
4	Transmission	87.14	80.00	82.86	87.14	92.86	LR

**Table 2:** Confusion matrix for all category samples.

Actual Class	Predicted Class						Sum
	Healthy	Mg Def.	B Def.	HLB-Symptomatic	Zn Def.	Zn Def. + HLB-Positive	
Healthy	50	0	0	0	0	0	50
Mg Def.	0	50	0	0	0	0	50
B Def.	0	0	40	0	0	0	40
HLB-symptomatic	0	0	0	50	0	0	50
Zn Def.	0	0	0	0	48	2	50
Zn Def. + HLB-positive	0	0	0	3	3	14	20
Sum	50	50	40	53	51	16	260

**Table 3:** Results of classification of the infection status of HLB.

Actual Class	Predicted Class		Sum
	HLB-Positive	HLB-Negative	
HLB-positive	67	3	70
HLB-negative	2	188	190
Sum	69	191	260

**Table 4:** GLCM-based texture features.

Feature	Equations	
Energy	$\sum_{i}\sum_{j}p{\left(i,j\right)}^{2}$	(8)
Correlation	$\sum_{i}\sum_{j}\frac{(i\cdot j)p(i,j)-\mu_{x}\mu_{y}}{\sigma_{x}\sigma_{y}}$	(9)
Homogeneity	$\sum_{i}\sum_{j}\frac{p(i,j)}{1+|i-j{|}^{2}}$	(10)
Contrast	$\sum_{i}\sum_{j}\left|i-j{|}^{2}p(i,j\right)$	(11)
Mean	$\mu =\frac{\sum_{p\in R}g(p)}{F}$	(12)
Standard deviation	$\sigma =\sqrt{\frac{\sum_{p\in R}{\left(g(p)-\mu \right)}^{2}}{F}}$	(13)
Dependency	$\mu_{x}=\sum_{i}i\sum_{j}p(i,j)$, $\mu_{y}=\sum_{j}j\sum_{i}p(i,j)$ $\sigma_{x}^{2}=\sum_{i}{\left(i-\mu_{x}\right)}^{2}\sum_{j}p(i,j)$, $\sigma_{y}^{2}=\sum_{j}{\left(j-\mu_{y}\right)}^{2}\sum_{i}p(i,j)$	

**Table 5:** Confusion matrix for the identification results of Zn-deficient + HLB-positive samples.

Actual Class	Predicted Class		Sum
	Zn Deficiency	Zn Def. + HLB-Positive	
Zn deficiency	48	2	50
Zn Def. + HLB-positive	3	17	20
Sum	51	19	70

## Data Availability

The datasets generated and/or analyzed during this study are available from the corresponding author upon reasonable request.
